# Conformational Changes in Surface-Immobilized Proteins Measured Using Combined Atomic Force and Fluorescence Microscopy

**DOI:** 10.3390/molecules28124632

**Published:** 2023-06-08

**Authors:** Cristian Staii

**Affiliations:** Department of Physics and Astronomy, Tufts University, Medford, MA 02155, USA; cristian.staii@tufts.edu

**Keywords:** proteins, protein dynamics, conformational changes, free-energy landscape, atomic force microscopy, fluorescence microscopy

## Abstract

Biological organisms rely on proteins to perform the majority of their functions. Most protein functions are based on their physical motions (conformational changes), which can be described as transitions between different conformational states in a multidimensional free-energy landscape. A comprehensive understanding of this free-energy landscape is therefore of paramount importance for understanding the biological functions of proteins. Protein dynamics includes both equilibrium and nonequilibrium motions, which typically exhibit a wide range of characteristic length and time scales. The relative probabilities of various conformational states in the energy landscape, the energy barriers between them, their dependence on external parameters such as force and temperature, and their connection to the protein function remain largely unknown for most proteins. In this paper, we present a multimolecule approach in which the proteins are immobilized at well-defined locations on Au substrates using an atomic force microscope (AFM)-based patterning method called nanografting. This method enables precise control over the protein location and orientation on the substrate, as well as the creation of biologically active protein ensembles that self-assemble into well-defined nanoscale regions (protein patches) on the gold substrate. We performed AFM–force compression and fluorescence experiments on these protein patches and measured the fundamental dynamical parameters such as protein stiffness, elastic modulus, and transition energies between distinct conformational states. Our results provide new insights into the processes that govern protein dynamics and its connection to protein function.

## 1. Introduction

Proteins are complex macromolecules that perform an extraordinary variety of biological functions ranging from gene regulation and catalysis of biochemical reactions to mechanical scaffolding of cells and mediation of signal transmission along inter- and intracellular pathways [1,2,3,4,5,6,7,8,9,10,11,12,13,14,15,16]. The vast range of protein functions is attributed to their high specificity for the molecules with which they interact, necessitating very accurate 3-dimensional (3D) protein structures. Although the static average structures for many proteins have been known for decades, it has only relatively recently become clear that protein functions are also closely tied to their physical motions. Proteins can undergo a large number of motions (conformational changes) around the average structure as a result of external factors, such as thermal energy fluctuations, interactions with other molecules, or binding of ligands. These conformational changes, which involve alterations in the protein’s 3D structure, are essential for various biological processes such as enzyme activity, protein–protein interactions, and signaling pathways.

Many biomimetic materials depend on protein biological functions for diverse applications, including the creation of biological scaffolds for tissue engineering or the patterning of protein microarrays for biosensing [17,18,19]. Ligand binding is one of the most important function of proteins, and their excellent molecular recognition capabilities make many natural as well as artificially designed proteins highly suitable for biosensing applications [16,17,18,19,20,21,22,23,24,25,26,27,28,29]. Protein-based biosensors are devices that detect the presence of a single molecular species (ligand) in complex mixtures by linking the proteins’ molecular recognition capabilities to a detection device via an appropriate signal transduction mechanism (e.g., chemical, mechanical, optical, or electrical signals). Therefore, a detailed understanding of protein conformational changes is crucial for both comprehending their biological function and applying this knowledge to developing practical biomaterials (such as biocompatible implants, biosensors, and biominerals).

Another challenge in biomaterials design is controlling protein adsorption and self–assembly on surfaces. For example, the ability to control the packing density, orientation, and type of exposed bioactive sites of the adsorbed protein layer is a key factor for the biocompatibility of medical implants [17]. Similarly, the ability to pattern protein microarrays, which comprise libraries of proteins immobilized in a 2-D addressable grid on a chip, is essential for biosensing and proteomics applications [18]. Although existing substrate-immobilization methods enable the preparation of thin protein films, these techniques often result in the loss of the native conformation and protein bioactivity. Developing new immobilization methods that preserve the bioactivity of the surface-attached proteins would represent a significant advancement in creating practical and viable biomaterials [17,18,19]. Moreover, transitioning from microscale to nanoscale offers numerous advantages: reduced sample size and quantities, more homogeneous patterns due to their smaller sizes, and higher resolution monitoring of specific protein interactions.

In this paper, we present a multimolecule approach for immobilizing proteins on surfaces and for measuring protein conformational changes. This method employs an atomic force microscope (AFM)-based patterning technique called nanografting to immobilize periplasmic binding proteins at specific locations on Au substrates. This technique makes it possible to precisely control the location and orientation of proteins on the substrate and to create ensembles of biologically active protein molecules, which self-assemble in nanoscale regions (protein patches) with well-defined boundaries on the Au substrate. Each patch contains a controllable number (100–1000) of protein molecules all oriented in parallel with respect to each other. To demonstrate the effectiveness of this method, we performed AFM–force compression and fluorescence measurements on the surface-immobilized protein patches. Our results show that the combination of AFM and fluorescence microscopy measurements offers exceptional capabilities for investigating proteins’ biomechanical properties and dynamics with high spatial, temporal, and force resolutions. We also explore the potential implications of utilizing these innovative techniques for measuring protein conformational changes and obtaining novel insights into the general dynamic mechanisms governing protein function. These results have significant impact for the fields of protein folding, proteomics, biosensing, and tissue engineering.

### 1.1. Protein Conformational Changes and the Free-Energy Landscape

**Figure 1:**
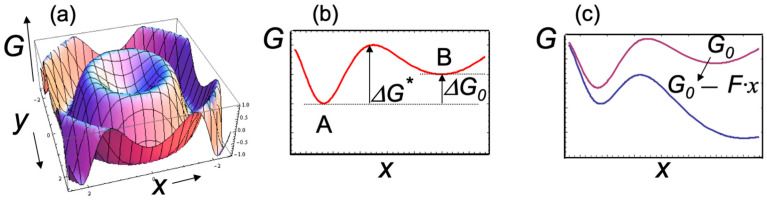
Schematic representations of the free — energy landscape. (**a**) The free energy *G* is shown as a function of two conformational coordinates *x* and *y*, while the full landscape might be determined by more than two coordinates. The actual landscape for a typical protein shows a very large number of local minima, corresponding to many slightly different conformations that a complex system can assume. For simplicity, a smooth surface with only two minima and one energy barrier is shown (scales on the axes are arbitrary). (**b**) Projection of the free-energy surface along the mechanical coordinate *x*. The figure shows two free—energy minima corresponding to two distinct observable states A and B, as well as the standard free energy Δ*G*_0_ and the energy barrier ΔG* of the transitions between the states. (**c**) An external force *F* > 0 (i.e., which tends to increase *x*) changes the free energy surface (from red to blue curve) with the net effect of increasing the forward transition rate and the population of state B.

Generally, the large ensemble of conformations that a protein can sample can be described by a multidimensional free-energy landscape, which defines the energy of different conformational states, their probabilities of occupation, and the energy barriers between them [1,2,3,4,5,6,7,8,9,10,11,12,13,14,15]. Conformational changes (protein motions) are then described as transitions between different conformational states in this energy landscape. Most proteins consist of multiple quasiindependent structural domains, with individual motions covering a wide range of time scales (from nanoseconds to seconds) and length scales (from fractions of an angstrom to several nanometers) [5,6,7]. The concept of a free-energy landscape is very useful for studying protein dynamics: at any given moment, a single protein molecule occupies a specific conformational state, while the conformational space that a protein can explore is described by a hypersurface (Figure 1) representing the free energy as a function of all the conformation coordinates [1]. In principle, the dimensionality of this hypersurface could be very large. However, in practice the protein has a relatively small number of subdomains that move independently, and therefore its conformational changes can be modeled with a small number of effective conformational coordinates. Different states are populated based on their free energy within this multidimensional landscape, while protein motions can be described as transitions between different conformational states [1,2,3,4,5].

Mechanical degrees of freedom (such as the end-to-end distance of a molecule when stretched or compressed, the linear or angular distance between two functional domains of the protein) represent well-defined conformation coordinates that are particularly useful for describing the effects of an applied external force on the free-energy surface. For example, Figure 1c shows that the main effect of an applied force *F* is to “tilt” the free-energy surface along the mechanical conformation coordinate such that [1]

(1) $$\Delta G_{0}-F\cdot \Delta x=-k_{B}T\cdot \ln K_{eq}(F)$$

where Δ*G*_0_ represents the standard state of free energy, Δ*x* is the change in the mechanical coordinate, *k_B_T* is the thermal energy, and *K_eq_*(*F*) is the equilibrium constant. An external applied force *F* can either support (*F* > 0) or oppose (*F* < 0) a transition, thus altering the relative populations of different conformational states (for example, states A and B in Figure 1). The kinetic rates of the transitions between states A and B are also modified by external forces. For example, the forward kinetic rate for the A → B transition is given by [1]:

(2) $$k_{A\to B}=k_{0}\cdot \exp[-(\Delta G^{*}-F\cdot \Delta x)/k_{B}T]$$

where ΔG* is the free-energy barrier of the transition, and *k*_0_ is a (force-independent) factor related to the characteristic frequencies in the potential well at state A. Equation (2) shows that the kinetic rates of any transitions, such as ligand-induced conformational changes, can be determined from their exponential dependence on the applied force along the mechanical reaction coordinate.

### 1.2. Atomic Force and Fluorescence Microscopy Measurements of Protein Conformational Changes

There is extensive literature on AFM-force spectroscopy, single fluorescence, and fluorescence resonance energy transfer (FRET) spectroscopy measurements on proteins, both at the single-molecule and bulk levels, and many excellent review papers are dedicated to these topics (see for example [30,31,32,33,34,35,36,37,38,39,40,41,42]). In this section, we will briefly summarize some of the most relevant results for our measurements. The innovative aspects of our approach compared to these methods will be outlined in Section 2 and Section 3.

Mechanical methods, such as AFM–force extension experiments [1,30,31,32] and optical tweezers [43,44,45] have been successfully employed for studying the folding/unfolding energy landscape of single-molecule proteins and other biomolecules. For example, the analysis of force–extension curves for RNA hairpin molecules has revealed the existence of critical forces associated with transitions between different folding states [43]. Direct measurements of the area under the force–extension curve around one of these critical values have been used to determine the free-energy associated with particular conformational changes [43]. Cecconi and collaborators have shown that the extension vs. time traces at constant force could be employed to determine the equilibrium constant for the transition between different folding states of the RNase H protein [44]. The plot of ln(*K_eq_*) vs. applied force in these experiments yields a direct measurement of the standard free energy of the transition (see Equation (1) above). Moreover, single-molecule AFM–force-clamp measurements have revealed distinctive “sawtooth-like” patterns in protein folding/unfolding experiments, which contain valuable information related to the unfolding pathways and mechanical stability of the molecules being studied [1,2,43,44,45]. Using the dynamics of single-molecule AFM–force experiments, researchers have also inferred the distribution of free-energy barriers [46,47], the presence of distinct folding intermediate states [44], and even the existence of critical (“glass-transition”) forces below which the states interconvert between local minima on multiple timescales [48]. 

Fluorescence resonance energy transfer (FRET) enables the measurement of protein conformational changes with very high temporal resolution (μs ms time scales) [38,39,40,41]. The main concept involves introducing two fluorescent probes (chromophores) at suitable sites of the protein. FRET occurs between the two chromophores when the emission spectrum of one (the donor) overlaps with the excitation spectrum of the other (the acceptor). A modification in protein conformation results in a change in donor–acceptor distance, consequently leading to a change in the FRET signal. This signal is highly sensitive to small changes in the acceptor–donor distance, and the statistics of the emitted photons (FRET intensity, efficiency, distribution of FRET signal) are related to the number of states explored by the molecules at μs time frames [38,39,40,41]. In recent years, several experiments have demonstrated the feasibility of both single fluorescence and FRET measurements on ensembles of proteins tethered to various surfaces [20,39,49]. FRET experiments work in these ensembles because the family of proteins utilized (periplasmic binding proteins; see below) have relatively large cross-sections (5–10 nm in linear dimensions), ensuring that FRET pairs from adjacent molecules are well separated. Another significant advancement has been the identification of a general strategy for identifying allosteric sites that undergo local motions in conjunction with global hinge–bending conformational changes while preserving protein bioactivity. For instance, Hellinga et al. have reported several methods for creating cysteine mutations at special sites, thus allowing for site-specific attachments of fluorophores [26,28]. Additionally, other studies have shown that combining AFM and fluorescence microscopy offers unique advantages, enabling optical images with lateral resolution significantly lower than the diffraction limit [50,51]. Nevertheless, the full potential of these techniques for imaging biological molecules remains to be fully explored. 

### 1.3. Periplasmic Binding Proteins

**Figure 2:**
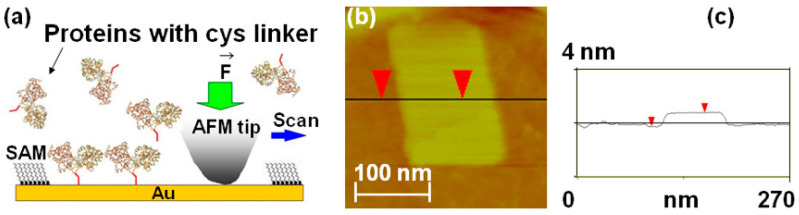
(**a**) Schematic of nanografting. MBP proteins have two domains of similar size linked by a central “hinge” region, which acts as a ligand binding site; upon binding, the maltose protein undergoes a large conformational change from an “open” (ligand-free) state to a “closed” (ligand-bound) state (the interdomain angle changes by 35°, and the relative distance decreases by 0.6 nm) [20]. For our experiments, we introduced a double cysteine linker (schematically shown here as a red tail) at the C-terminus of the MBP. The dicys linkers (red tails in the figure) bind covalently to Au via their thiol group. The figure also shows that the ligand binding site (top of the protein) is oriented toward the solution after nanografting. (**b**) AFM topographic image of a nanografted dicys-MBP protein patch. The surrounding PEG layer is formed by HSC_11_-EG_3_ (SAM) molecules that self-assemble on the Au surface. (**c**) Cross-sectional line scan corresponding to the horizontal line in image (**b**). The measured difference in height between the MBP and the surrounding PEG layer is Δ*h* = 1.1 nm.

The periplasmic binding protein (PBP) family contains about 100 distinct protein types that function as high-affinity receptors for nutrient transport across the inner membrane of bacteria [20]. Almost all PBPs share a fundamental two-domain structural fold consisting of two similarly sized lobes (domains) linked by a flexible “hinge” region, which serves as a binding site for various ligands (Figure 2a) [20,21,22,23,24,25,26,27,28,29]. These proteins are known to adopt two very distinct conformations: a ligand-free (open) state and a ligand-bound (closed) state. Ligand-binding events induce relatively large (~0.6 nm), reversible conformational changes (open–closed transitions) between these two states [20]. Furthermore, PBPs display a very specific recognition for a large class of analytes (various sugars, amino acids, metals ions etc.) and have been studied in the context of biosensing applications [20,28,29]. Several PBSs such as maltose-binding protein (MBP), glucose-binding protein (GBP), and ribose-binding protein (RBP) have been well characterized at the structural, biochemical, and molecular level [20,21,22,23,24,25,26,27,28]. MBP, in particular, has been studied with NMR [42] electrochemistry [52], single-molecule fluorescence, and FRET [20,29,53,54] measurements, making it one of the best-characterized systems for testing novel methods to probe the protein free-energy landscape. Several fluorescence and FRET studies have also been performed on GBP and RBP [29]. As far as we know, there are no systematic AFM studies on PBPs. Furthermore, all previous studies on PBsP have focused on the two state (open–closed) transitions, whereas the actual transition pathway, the existence of possible intermediate conformational states, and the energy barriers between them are unknown. A related family of proteins consist of P-glycoproteins (PGPs). These are transmembrane proteins members of the ATP-binding cassette (or ABC transporter family), a group of proteins homologous to bacterial PBPs, which couple the energy released from ATP hydrolysis to the translocation of a wide variety of substances into or out of cells [55]. PGPs have been found to reduce the effective concentration of chemotherapeutic drugs inside cells, and it is hypothesized that this drug-resistant property can be attributed to protein conformational changes upon binding various nucleotides (ATP, MgATP) [55]. Despite their significant biological role, very little is known about conformational changes and the dynamics of PGPs. The novel multimolecule techniques presented in this paper can be employed to measure conformational changes and to map the free-energy landscape in PBPs and PGPs by nanografting these proteins onto Au surfaces and and measuring their dynamics through AFM–force compression and combined AFM–fluorescence experiments.

## 2. Results and Discussion

### 2.1. Maltose-Binding Protein Nanografted on Au Substrates

Maltose-binding proteins (MBPs) were bioengineered to contain a double cysteine linker (dicys–MBP) (Materials and Methods). Next, these proteins were immobilized in small (order of 10 to 100 nm) patches at well-defined locations on Au substrates using nanografting (Figure 2b).

Nanografting is an AFM-based nanolithography technique that consist of three main steps: (1) a self-assembled monolayer (SAM) of a thiol-terminated molecule that resists protein adsorption (such as undecanethiol triethylene glycol, HSC_11_-EG_3_, or PEG for short) is deposited onto the Au substrate, and a flat region of this surface is selected through low force AFM-imaging; (2) a small area of the selected region (~100 nm × 100 nm^2^) is scanned at a relatively high load to catalyze the exchange of the PEG molecules from the original SAM with the cysteine-terminated proteins present in the adjacent buffer solution (the cysteine linker binds covalently to Au via its thiol group; Figure 2a); and (3) the outcome of this process is the formation of a nanografted “protein patch” which can then be imaged at low applied forces via AFM topography measurements (Figure 2b). We employed standard recombinant DNA techniques [56,57,58,59,60,61,62] to insert a double cysteine (dicys) residue at the C-terminus of MBPs (the dicys linker is illustrated schematically as the red tail in Figure 2a). The inserted dicys had a dual role: first, it provided a unique site for protein immobilization, and second, it ensured that the dicys–MBP molecules had only one possible orientation on the Au substrate (nanografting replaced the sulfur–gold interaction of the surrounding alkanethiol SAM with a sulfur gold interaction involving the dicysteine linker of the protein). This configuration was also designed to orient the ligand (maltose)-binding site of the MBP toward the buffer solution (Figure 2a). The measured difference in height between the MBP and the surrounding PEG layers was 1.1 nm (Figure 2c). Given that the height of the PEG monolayers on Au is 2.3 nm [56,57], the total height of the dicys-MBP patch was 3.4 nm, in excellent agreement with protein dimensions reported in literature [20,35,36]. In previous work, we confirmed the biochemical activity of the nanografted proteins through in situ AFM friction measurements, when sugar (maltose or maltotriose) was added to the buffer solution. One of the main results of this earlier work was the demonstration that the immobilization process, the spatial confinement associated with the surrounding proteins, and the protein–substrate interactions do not alter the protein function and ligand-binding activity [56,57]. In the present study, we performed AFM compression experiments of nanografted MBP patches without introducing any ligand in the solution. In the following sections, we present the application of this multi–molecule approach for probing conformational changes in MBP proteins and for measuring their biomechanical parameters and structural stability.

### 2.2. AFM–Force Compression Measurements of MBPs 

**Figure 3:**
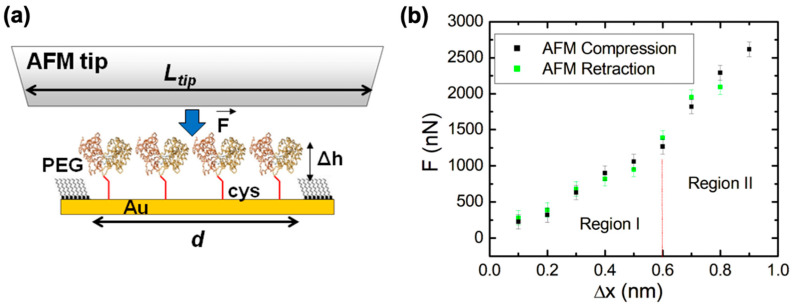
(**a**) Schematic of the AFM–force compression experiments performed on a nanografted dicys–MBP protein patch. The height of the nanografted MBPs is larger than that of the surrounding PEG by Δ*h* = 1.1 nm (Figure 2c). The AFM tip has a flat area larger than the patch size, and thus all proteins are simultaneously compressed by the tip (**b**) Plot of the applied force *F* vs. change in the height of the protein patch (deformation) Δx measured for (i) the AFM tip approaching the gold substrate (i.e., compression, shown by black data points) and (ii) the AFM tip moving away from the substrate (i.e., retraction, shown by the green data points). Error bars represent the standard error of the mean resulting from the AFM–force calibration measurements (see Materials and Methods). The compression and retraction data points are very close, demonstrating that the compression is reversible in the range Δx from 0 to 0.9 nm. The significance of the two regions shown in the graph is discussed in the main text. The work performed by the AFM tip during compression equals the area of the region under the curve containing the data points (see main text). The protein patch is irreversibly damaged for compression values Δx>0.9 nm (corresponding to external forces: *F* > 2600 nN).

We conduct AFM–force compression experiments on protein patches to investigate the fre- energy landscape of surface-immobilized dicys–MBPs (Figure 3). As discussed above, an external force “tilts” the energy landscape, subsequently changing the protein’s conformation and transition rates (Figure 1 and Equations (1) and (2)). Figure 3a shows the schematics of the AFM–force compression experiments. The uncompressed dicys–MBP proteins have a larger height than that of the surrounding PEG (the height difference is Δ*h* = 1.1 nm, as shown on Figure 2c). The linear size of the nanografted protein patches is in the range *d* = 50 to 150 nm. To perform force compression measurements on the entire protein patch, we used a different type of AFM tip than the one used for nanografting (see Materials and Methods and references [63,64,65]). The tip used for compression experiments had a large radius of curvature (R~6000 nm), making it locally flat with linear dimension *L_tip_* (Figure 3a). In the region where the AFM tip compressed the protein patch, we closely approximated that *L_tip_ >> d.* This ensured that all proteins in the patch were simultaneously compressed by the flat AFM tip (Figure 2a).

Figure 3b shows an example of the data (compression curves) acquired during AFM–force compression experiments performed on the protein patch shown in Figure 2b. The compression of the protein patch Δx (defined as the negative of the change in the patch height) increased with the increase in the force applied by the AFM tip, up to a maximum value of Δx≈0.9 nm obtained for a maximum compression force $F_{max}\approx 2600$ nN (black data points in Figure 3b). The compression curve was reversible, as shown by the green data points in Figure 3b: these data points represent the compression values Δx as the applied force is *decreased* from $F_{max}$ to 0 nN. The protein patch is irreversibly damaged if the AFM applied force *F* is increased above the maximum value $F_{max}\approx 2600$ nN. This maximum compression force is related to the overall structural stability of the proteins as we discuss in Section 2.4 below. We also note that during the compression experiments, the change in height of the protein patch was less than the height difference between the patch and the surrounding PEG layer; that is, Δx<Δh (Figure 3a). Therefore, the AFM tip compresses only the nanografted dicys–MBP patch and *not* the PEG layer. Given the lateral dimensions of one MPB molecule of 4 × 6.5 nm (taken from crystallographic data [20]) and the overall area of the nanografted protein patch measured with the AFM, ΔA≈16,590 nm^2^ (Figure 2b), we could calculate a total number N=638 of proteins simultaneously compressed by the AFM tip. The area under the compression curves in Figure 3b is equal to the total work performed by the AFM tip: $W\approx 961\times 10^{-19}$ J. We discuss the implications of these results for advancing our knowledge of conformational changes and the biomechanical properties of MBP in Section 2.4.

### 2.3. Fluorescence Microscopy Measurements of Nanografted Protein Patches 

**Figure 4:**
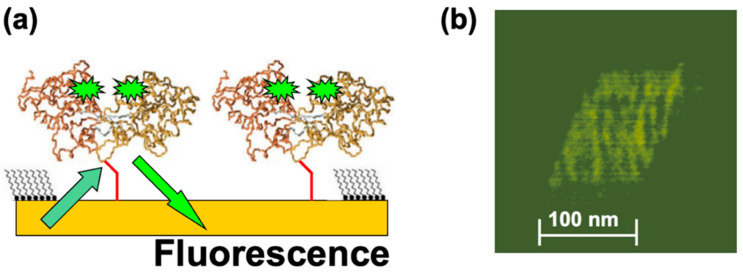
(**a**) Schematic of the fluorescence experiments. Alexa 488 fluorophore pairs are attached at allosteric sites on the MBP proteins. The distance between the fluorophore pairs inside the protein is much less than distance between the two adjacent nanografted proteins, so we expect negligible interaction between pairs belonging to two different proteins. (**b**) Fluorescence image acquired from the nanografted MBP patch. The variation in fluorescence intensity does not represent single-molecule resolution.

In a different set of experiments, MBP proteins were functionalized with fluorescent markers (Alexa 488 fluorophores; see Materials and Methods) and then nanografted on the Au substrate. Figure 4a shows the schematic of the fluorescently modified dicys–MBP immobilized on Au. Figure 4b shows an example of the fluorescence image obtained from the nanografted protein patch. 

The fluorescent image was acquired in small time frames (each frame is of the order of 1 s). However, the observed variation in the measured fluorescence intensity does not necessarily imply single-molecule resolution. There are several other factors that could have led to the observed variation in fluorescence intensity, such as protein–protein interactions and substrate quenching [41]. These factors will be investigated in future experiments, as discussed in Section 4 below. These results demonstrate that it is possible to combine the precise positioning of proteins on substrates and control over their orientation allowed by nanografting with the high spatial (AFM imaging) and temporal (fluorescence) resolutions. Previous experiments performed on several mutants of periplasmic binding proteins, including MBPs, have shown that [20,29] (i) the protein ligand-binding properties are minimally affected if fluorophores are attached at specific allosteric sites, (ii) the fluorescence intensity of these conjugates changes cooperatively with respect to ligand binding, and (iii) changes in fluorescence intensity affect ligand binding in a concentration-dependent manner. Moreover, previous work demonstrated that the fluorescence lifetime and efficiency is greatly improved due to protein–substrate quenching interactions in systems with ensembles of proteins compared to single-protein experiments [29,49,55]. Although the exact mechanism of the observed increase in fluorescence intensity is not completely understood, it has been suggested that several factors could contribute to this effect: surface screening by multiple proteins located in close proximity to each other, decrease in the effective extinction coefficient for the fluorophore, or increase of the acceptor–donor overlap integral for FRET pairs [49]. To our knowledge, our results represent the first fluorescent images obtained for protein patches immobilized at well-defined locations on substrates via nanografting. Together with the AFM–force compression measurements, these results demonstrate that combined AFM—fluorescence microscopy experiments carried out on nanografted protein patches provide a powerful approach for measuring conformational changes in proteins with high spatial and temporal resolutions. 

### 2.4. Discussion

The results presented in the previous sections demonstrate the strength of a multimolecule approach combined with nanografting for studying the conformational changes in proteins. To summarize, the results show that it is possible to (a) carry out protein immobilization and perform all measurements in a controlled environment (protein buffers) such that the proteins will retain their folding conformation and therefore their bioactivity; (b) investigate nanoscale regions (protein patches) with well–defined boundaries, each patch containing a controllable number of proteins (100–1000), and having all molecules oriented in parallel; (c) form highly ordered, defect-free protein SAMs, particularly suitable for studying protein conformational changes induced by external stimuli (ligand binding, external forces, etc.); (d) perform precise, high resolution measurement of single-molecule quantities (protein orientation in different states, transition energies; see below); and (e) have precise control over the direction and magnitude of external forces while minimizing the nonequlibrium effects associated with external perturbations of the molecular system.

Figure 3b shows reversible force vs. compression (F vs. Δx) curves for nanografted dicys–MBP for AFM applied forces in the range of $0\leq F\leq F_{max}\approx 2600$ nN. These curves display two distinct regions: region I (corresponding to *F* between *0* and $F_{1}=1260$ nN, and compression Δx between 0 and $\Delta x_{1}=0.6$ nm) and region II (corresponding to $F_{1}\leq F\leq F_{max}$ and compressions $\Delta x_{1}\leq \Delta x\leq \Delta x_{max}=0.9$ nm). For forces above value $F_{max}\approx 2600$ nN, irreversible damage of the patch occurs. The slopes of the *F* vs. Δx curve represent the stiffness *K* of the protein patch. From the data in Figure 3b, we obtain the following: $K_{1}=2110$ N/m (for region I), and $K_{2}=4503$ N/m (for region II). Since the patch contains N=638 protein molecules, we can calculate the stiffness for an *individual* dicys–MBP protein molecule as follows: $k_{1}=3.3$ nN/nm (region I) and $k_{2}=7.1$ nN/nm (in region II). Moreover, by modelling each protein as an elastic rod with length *h* = 3.4 nm (protein height) and cross-sectional area a=26 nm^2^ (for crystallographic data, see Section 2.2), we obtain the following estimate for the protein Young’s modulus: $E=\frac{k\cdot h}{a}$, from which we obtain $E_{1}=430$ MPa (region I), and $E_{2}=928$ MPa (region II).

In our previous work [56,57], we performed AFM compression experiments on nanografted MBP proteins when ligand (maltose or maltotriose) was added to the solution. However, in this previous work, we used AFM cantilevers with sharp tips (tip radius 15–20 nm), and therefore only a small region of the protein patch was compressed by the AFM. This resulted in relatively large uncertainties in the estimated tip–protein patch contact area and consequently in large uncertainties in the total number of proteins compressed by the AFM tip for any given force curve. In the current work, by using a flat AFM tip (Figure 3a), we could compress the whole protein patch. Therefore, this approach allows us to estimate with high accuracy the total number of proteins compressed by the AFM tip and to calculate the stiffness and Young’s modulus for individual dicys–MBP proteins. To our knowledge, this is the first time these two important biomechanical parameters (stiffness and Young’s modulus) have been measured for any type of PBP. The values of elastic moduli reported in literature for other types of proteins are mostly computational and vary between several hundred MPa to tens of GPa [58]. Our measured values are therefore in the range of the elastic modulus reported for other types of proteins.

To understand the origin of the two regions in the force–compression curves, we calculate the mechanical work performed by the AFM tip during compression. The work is equal to the area under the curve W=∫F · dx. Therefore, for region I we obtain $W_{1}\approx 379\;\times \;10^{-19}$ J, while for region 2, we obtain $W_{2}\approx 582\;\times \;10^{-19}$. Again, by dividing with the total number of proteins compressed by the tip (N=638), we obtain the compression work/protein in the two regions, $w_{1}\approx 0.59\;\times \;10^{-19}$ J/protein (region I) and $w_{2}\approx 0.91\;\times \;10^{-19}$ J/protein (region II), respectively. We note that the values of the work/protein in region I is very close to the transition energy between the open and the closed states of the MBP reported in the literature from NMR or calorimetric measurements [14,20,26]: $\Delta G_{0}\approx 0.58\;\times \;10^{-19}$ J. Thus, we can conclude that during the AFM compression in region I, the two lateral domains of the MBP protein (Figure 3a) are brought together such that the protein transitions from the open to the closed state. As can be seen in Figure 1b,c, the effect of the AFM compression is to produce an overall “tilt” of the free-energy landscape of the dicys–MBP, such that the closed states (state B in Figure 1b) have a final free energy that is *lower* than the free energy of the open states (state A in Figure 1b). This “tilt” in the free energy is represented schematically as the shift from the red to the blue curve in Figure 1c. Thus, the net effect of increasing the external force applied by the AFM from 0 to $F_{1}=1260$ nN is to induce the transition between open (A) to closed (B) states for the nanografted dicys–MBP protein molecules. The value for the maximum protein compression $\Delta x_{1}=0.6$ nm in region I is also consistent with the distance change between the MBP interdomains during the open–closed transition [20] (see also the caption to Figure 2).

In region II, the proteins are further compressed until Δx=0.9 nm and $F_{max}\approx 2600$ nN. The total mechanical work (total area under the curve in region I + region II) performed by the AFM tip per protein is $w=w_{1}+w_{2}\approx 1.5\;\times \;10^{-19}$ J/protein. This value is very close to the total energy required to denature the MBP proteins by breaking the bonds between the two lateral domains that keep the MBP molecule stable. This energy was previously measured by calorimetry and fluorescence experiments [14,20,26]. This result explains why applying a force larger than $F_{max}$ (and therefore a compression larger than 0.9 nm) results in the irreversible damage of the nanografted patch: the AFM tip denatures the proteins by applying mechanical energy that overcomes the energetic cost required to keep the protein stable. We emphasize that, quite remarkably, these two results—measurement of open–closed transition energy and the energy required to denature the protein—are obtained solely from AFM–force compression experiments on nanografted protein patches. Therefore, these results demonstrate the proof of principle for the future experiments discussed below.

## 3. Materials and Methods

### 3.1. Sample Preparation

The process of adding cysteine residues to MBP involves inserting the designed PCR primer into the corresponding binding domain (e.g., *E. coli* maltose-binding domain for MBP) and expressing this fragment into the purification vector as described in the literature [57,58]. The dicys–MBP proteins are expressed in *E. coli* and were purified using a method that employs self-cleaving elastin-like polypeptide (ELP) fusion tags, which has been described in detail previously [60,62]. The high-throughput method (~100 mg proteins/L of cell culture) that resulted from this approach was crucial for the high success rate of the reported nanografting experiments. Alexa fluorescence markers were attached to the protein by employing well-established protocols [20,29,62], that use the covalent binding of the fluorescent label to the corresponding natural amino-acid residue via the corresponding cofactor chemistry [40,41]. 

The substrates were prepared through thermal evaporation of Au on mica substrates in a vacuum chamber (K.J. Lesker Co., EJ1800 Bell Jar, Jefferson Hills, PA, USA) at a pressure of 10^−7^ mbar and a temperature of 300 °C. After evaporation, the transparent (up to a few tens of nm thick) film of Au was immersed into a 0.1 mM solution of alkane-thiol HSC_11_-EG_3_ (Sigma-Aldrich, St. Louis, MO, USA). These thiol-terminated molecules formed a uniform, protein-resistant SAM on the Au surface. The surface was imaged with the AFM before the nanografing procedure.

### 3.2. AFM Imaging, Nanografting, AFM-Compression and Fluorescence Measurements

Nanografting, AFM, and fluorescence experiments were performed on an Asylum Research MFP–3D–BIO AFM (Asylum Research, Santa Barbara, CA, USA) integrated with an inverted Nikon Eclipse Ti optical microscope (Micro Video Instruments, Avon, MA, USA). This high-performance system combines molecular resolution AFM imaging with very high force sensitivity (pN) and the power of fluorescence imaging. By using an inverted optical microscope (Nikon Ti-E) as the AFM stage, the instrument allows for the correlation of AFM topography and force mapping with fluorescence with high precision and accuracy. The AFM was equipped with a fluid cell, a vibration isolation box, a Petri dish sample holder, and a bioheater, such that during the nanopatterning and measurement process, proteins were kept in their native buffer solution. Fluorescence measurements were performed on the inverted Nikon Eclipse Ti optical stage integrated with the Asylum Research AFM using the 60 X objective. The optical stage was equipped with a fiber-optic cable coupled to a CCD camera.

Nanografting and AFM imaging of the protein patches were carried out with commercially available SiN cantilevers (Bruker AFM Probes, Camarillo, CA, USA) with a nominal spring constant of 2.8 N/m. AFM–force compression measurements were performed using NP-O10 SiN probes with a spring constant of 15 N/m and spherical tips with a nominal radius of R = 6 μm (Bruker Corporation, Billerica, MA, USA). The AFM—compression measurements on protein patches were similar to the AFM measurements that were performed to measure the biomechanical properties of neuronal cells [64,65]. The same cantilever was used for all force–distance measurements. Before each measurement, the cantilever was calibrated on the Au substrate in the sample medium. The cantilever deflection sensitivity and spring constant were calibrated before each measurement. To limit energy dissipation due to viscoelastic effects, the indentation frequency was 0.5 Hz for all measurements. A typical force point has a fitting error of ≤20%, and the values obtained for the elastic modulus at every point were reproducible within this error. 

## 4. Future Perspectives and Conclusions

Understanding the response of proteins to external forces and their mechanical stability is crucial for a detailed description of protein conformational changes and the energy landscape. However, a comprehensive depiction of these biomechanical parameters remains elusive. Earlier single-molecule force spectroscopy experiments (see Section 1) have provided essential information about the free-energy surface of the folding/unfolding events for many proteins. Nonetheless, even the very high force resolution (fraction of nN) offered by current AFM instruments is not enough for the direct observation of most conformational changes of folded proteins at the *single-molecule* level [1,2,3,4,5,6,7,8,9,10,11,12,13,14,15,16]. Furthermore, one of the main problems to be overcome in these experiments is that a time-varying external force actively perturbs the molecular system, which leads to significant nonequilibrium effects and hysteresis. Finally, single proteins are anisotropic systems and therefore force-driven transitions might be dependent on the actual protein orientation when a force is applied [34,35,36]. Our results show that one can address these problems by performing AFM–force and spectroscopy measurements on *ensembles* of proteins (100–1000 molecules) *all oriented in parallel* on the Au substrate via nanografting. The proposed method is to start with the proteins in a state of stable local equilibrium (e.g., open or closed states) and to investigate the surrounding local minima and the transition energy separating them by generating force-induced conformational changes. A main advantage of this multimolecule approach compared to the single-molecule studies is that since the protein number and total mass are larger, the fluctuations and nonequilibrium effects due to time-varying external perturbations of the molecules are smaller [33,34,57,58]. Second, the pN/molecule level resolution of forces applied by the AFM is at least one order of magnitude smaller than are the forces required to induce conformational changes in ensembles of molecules [5,6,7,34]. Therefore, controlled measurements on ensembles of proteins will provide several net advantages over the single-molecule approach (fewer fluctuations, uniform compression forces, higher signal/noise ratio), allowing researchers to perform detailed investigations of the free-energy landscape. Thirdly, multimolecule measurements are easier to perform in buffer solutions since they do not require molecular resolution in positioning the AFM tip, and therefore problems related to thermal/mechanical drift of the cantilever are minimized. In addition, anisotropy and stochastic effects are dramatically reduced since the molecules have a well-controlled orientation on the substrate. Finally, we point out that many of the advantages of single-molecule measurements (absence of ensemble averages, control over the direction and magnitude of the applied forces) are also maintained in the nanografting experiments due to the parallel orientation of molecules immobilized at addressable, defect-free areas. 

Nanografted protein patches can also be used to perform combined AFM and fluorescence measurements on ensembles of proteins. Fluorescence measurements provide real-time information of the ligand- and force-dependent dynamics of fluorescently tagged proteins. Our results show that the energy landscape of MBPs around their equilibrium (open, closed) states are not altered significantly either by lateral protein–protein interactions within the nanografted patches or by the surface forces due to the Au layer [57]. In future experiments, we propose to measure the change in fluorescence intensity for fluorophore-tagged MBPs and other periplasmic binding proteins and to correlate this change with the measured variations in force, height, and binding kinetics measured with the AFM. 

**Figure 5:**
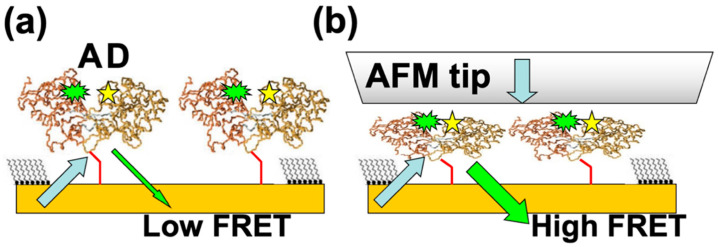
(**a**) Schematic of the proposed FRET experiments. Acceptor (A) and donor (D) pairs are attached at allosteric sites inside proteins; the distance between the A–D pair inside the protein is much less than distance between the two adjacent nanografted proteins, resulting in a negligible interaction between A–D pairs belonging to two different proteins. (**b**) AFM compression brings the FRET pairs together which results in a distance-dependent increase in the FRET signal.

Another very promising direction of research is to bioengineer fluorescence resonant energy transfer (FRET) acceptor–donor pairs at allosteric sites on the proteins [28,29,38,39,40,41]. Previous work has shown that the FRET efficiency in these systems could reach 75% [49]. We propose to nanograft FRET-labelled proteins on surfaces, so one can perform simultaneous combined AFM–FRET measurements on the protein patches (Figure 5). For different conformational changes induced by externally applied AFM–forces, one will record (a) the variation of the acceptor (donor) fluorescence intensity with time, (b) traces of FRET efficiency vs. time, and (c) histograms of the occupancy times in each conformational state. These data will provide details about force-induced conformational changes with high temporal resolution, therefore allowing the identification of higher resolution features of the free-energy landscape, such as the intermediate states and kinetic pathways that are time-averaged in the relatively slower AFM–force compression measurements. The correlation of high temporal resolution FRET with simultaneous high resolution (sub-nm and sub-nN) AFM–force measurements will therefore offer a very accurate multidimensional (distance, time, force) picture of conformational changes.

We emphasize that this novel approach offers unique advantages for measuring the protein free-energy landscape. First, we anticipate that the parallel orientations of nanografted molecules will greatly simplify the interpretation of the acquired spectra in both single fluorescence and FRET experiments. Second, large conformational changes will be induced by external forces applied via the AFM in a well-controlled way, and therefore these forces will be used to synchronize conformational changes, thus providing synchronized time trajectories for FRET measurements. Since the FRET signal intensity increases proportionally with the number of molecules [38,39,40], we expect to obtain FRET intensities tens to hundreds of times larger than those observed in single-molecule experiments. Thirdly, we note that nanografting can be used to immobilize ensembles of different types of proteins at specific locations on surfaces. By keeping these proteins in controlled environments, we anticipate that researchers will be able to reproduce some of the features that resemble the crowded environment found inside cells in vivo [3]. Therefore, we expect that this multimolecule approach is likely to be very relevant for investigating specific functions, such as ligand binding, as well as for biosensing and biocompatibility applications. Furthermore, we note that most fluorescence and FRET studies on PBP reported in the literature have been performed with individual proteins tethered on various solid surfaces [20,28,29], which demonstrate that for PBPs, the protein–substrate interactions play a minor role on the protein’s conformational dynamics or on the measured fluorescence/FRET signals.

We anticipate that these techniques will have significant impact for creating novel protein-based biomimetic and biosensing devices. Nanografting is a technique that avoids solvents and conditions that can alter the protein function (which might occur in other techniques such as microcontact printing for example). Therefore, nanografting is a highly effective method for patterning multifunctional devices for biosensing applications, as it enables the sequential immobilization of proteins with distinct functionalities at separate addressable locations on the same substrate. Single-fluorescence and FRET responses (calibrated by AFM measurements) can offer a general signal-transduction mechanism that reports ligand binding in a concentration-dependent manner for these protein-based biosensors. These techniques can also be applied to investigate the energy landscape in other types of biomolecules (such as DNA and RNA), measure conformational changes in protein-folding and unfolding experiments, and examine the underlying mechanisms that lead to alteration in the function of proteins due to changes in their native conformations.

## Data Availability

The data presented in this study are available within the manuscript.

## Sample Availability

Samples of the protein compound are available from the authors.
